# Efficacy of Abemaciclib in the Management of Refractory Metastatic Extramammary Paget’s Disease

**DOI:** 10.7759/cureus.27534

**Published:** 2022-07-31

**Authors:** Gary W Takahashi, John Vetto, Justin J Leitenberger, Arthur Hung

**Affiliations:** 1 Hematology & Medical Oncology, Oregon Health & Science University, Portland, USA; 2 Surgical Oncology, Oregon Health & Science University, Portland, USA; 3 Dermatology, Oregon Health & Science University, Portland, USA; 4 Dermatologic Surgery, Oregon Health & Science University, Portland, USA; 5 Radiation Oncology, Oregon Health & Science University, Portland, USA

**Keywords:** cutaneous adenocarcinoma, cyclin d kinase inhibitors, targeted therapy, abemaciclib, extramammary paget's disease

## Abstract

Published systemic therapy options for metastatic extramammary Paget's disease have largely been anecdotal due to the rarity of this disease, which has precluded the ability to conduct clinical trials. We describe the favorable response of a 72-year-old man with extramammary Paget's disease, whose disease has been controlled with the CDK4/6 inhibitor, abemaciclib. The rationale behind the selection of this therapy is discussed.

## Introduction

Extramammary Paget's disease (EMPD) is a rare generally indolent cutaneous adenocarcinoma arising from the apocrine glands of the epidermis that often presents as an eczematous lesion in the anogenital region. Histologically, the lesion resembles Paget’s disease of the breast. In localized disease, management is surgical, and wide local excision of the primary lesion is the standard of care, either via Mohs micrographic surgery, preferred due to lower recurrence rates with this technique, or wide local excision with 3-5 cm margins [1]. Adjuvant radiation therapy to nodal draining sites may decrease recurrence. However, when there is evidence of distant spread, systemic therapy is often recommended. The literature concerning active therapies is largely anecdotal, and published experience with antineoplastic agents has been limited by the rarity of this condition. Here we present a case of successful long-term control with the cyclin D kinase 4/6 inhibitor, abemaciclib.

## Case presentation

A 72-year-old man with a prior history of bladder cancer and chronic renal insufficiency resulting from previous cisplatin exposure presented with a suspicious pigmented lesion in the left inguinal crease. Biopsy revealed extramammary Paget's disease, both in situ and invasive. He underwent Mohs micrographic surgery to obtain negative margins on the groin, thigh, and the scrotal sac, and then had resection of the main tumor mass as well biopsy of a sentinel node and the Cloquet’s node in the operating room. Pathology revealed that the nodes were negative for metastasis, and the specimen was confirmed to contain invasive EMPD.

The patient was well until the following year, when examination and CT imaging revealed an enlarged left inguinal lymph node; fine needle aspiration revealed EMPD. He underwent lymph node dissection, and metastatic EMPD was found in several of the resected lymph nodes. He then underwent adjuvant radiation therapy, with delivery of 59.4 Gy photons to the left groin nodal basin. He remained without evidence of active neoplasm until six months later, when a surveillance CT scan revealed increase in the size of a left common iliac lymph node as well as a nodule anterior to the left iliac spine, as well as increasing left supraclavicular lymphadenopathy. Fine needle aspiration of a supraclavicular node confirmed the involvement by EMPD.

After a review of the literature, and because of the patient’s pre-existing renal insufficiency, he was treated with weekly paclitaxel, to which the lymphadenopathy improved significantly, but at the cost of increasing peripheral neuropathy. A review of his biopsy material revealed the expression of androgen receptor, which has been identified previously in EMPD tissue [2]. Endocrine therapy with the anti-androgen agent bicalutamide had been reported to produce several months of stable disease in an anecdotal case report [3]. Therefore, the patient was prescribed bicalutamide as maintenance therapy, hoping to suppress or delay recurrence after the improvement seen with paclitaxel. However, after seven months of bicalutamide, there was evidence of increasing nodal progression, and with the lack of better alternatives, he was treated with paclitaxel again. Four months after his last dose of paclitaxel, he developed symptomatic bony metastatic disease in the right iliac crest, for which he was given 30 Gy of radiation therapy.

Because his disease-free interval after paclitaxel pretreatment was brief, and his renal disease and symptomatic peripheral neuropathy made other options, such as platinum agents, unfeasible, we considered other agents. The tumor did not exhibit evidence of mismatch repair deficiency, and expression of PD-L1 TPS (22C3) was < 1%, so an immune checkpoint inhibitor therapy was not considered, as it was not clear that there was any expectation of benefit that would exceed toxicities associated with this approach. NexGen sequencing did not identify any actionable driver mutations.

After further reviewing the literature for therapy suggestions, we found a 2019 paper from Urata et al. [4] that reported that almost all specimens of EMPD they tested were positive for CDK4 overexpression. The patient was therefore recommended to begin abemaciclib at 100mg BID as a starting dose. This drug was selected over alternative CDK4 inhibitors due to its stronger inhibition of CDK4, which renders it active in breast cancer as monotherapy without the need for concomitant adjunct hormonal therapy. Excretion of this drug is primarily hepatic and could be given in the setting of his class IV chronic renal insufficiency, and 100 mg BID was selected as a starting dose.

**Figure 1:**
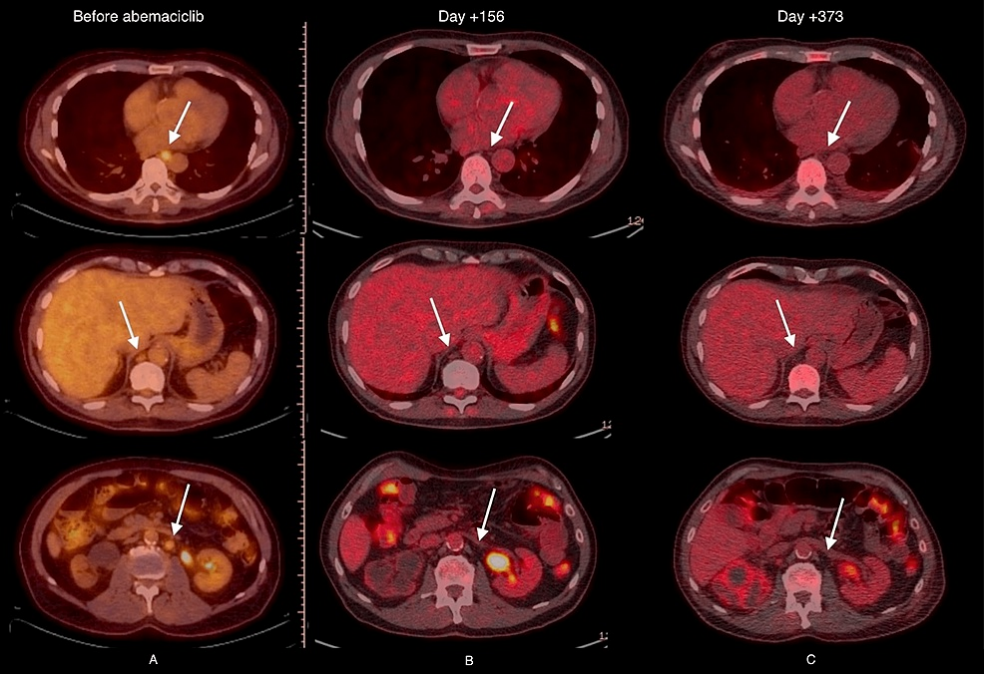
A) FDG-PET imaging obtained prior to administration of abemaciclib; B) imaging at day +156, indicating response; C) imaging at day +373, indicating durability of response.

The patient maintained remission status for 11 months, with no serious adverse effects or radiographic evidence of disease progression or recurrence as determined by serial examinations and PET/CT scans (Figure 1). 

However, eventually, his lesions recurred, and he required involved site radiation therapy to a bony metastatic site at the right ilium, which felt to be at risk of weight-bearing fracture. Metastatic sites which appeared in other locations remained asymptomatic and clinically inconsequential, and the decision was made to continue abemaciclib as it was felt that it was having continued clinical response. Eventually, due to intercurrent disease, his renal failure worsened to the point of his requiring dialysis support, which he declined, and the patient expired two and a half years after initiating abemaciclib therapy.

## Discussion

Extramammary Paget's disease is a rare adenocarcinoma arising out of apocrine glands or keratinocyte stem cells in the perineum but also, rarely, in the axilla. EMPD shares some features with breast cancer, such as positivity for BRST2 and GCDFP-15, as well as estrogen and androgen receptor expression. Median survival in the metastatic setting is approximately 1.5 years, with a 7% five-year survival [5]. The limited number of effective systemic chemotherapy options that have been identified is likely a factor. Although anti-estrogen therapy is of unquestioned benefit to patients whose breast cancer expresses estrogen receptors, the published experience with estrogen or androgen blockade suggests that the duration of benefit with this approach is limited to a few months. Various regimens involving cytotoxic chemotherapy have been described. As with breast cancer, taxanes, both paclitaxel and docetaxel, have been identified as active, either as monotherapy or combined with a platinum agent or anthracycline or fluoropyrimidine [5]. Although there are reports that document efficacy in the short term, the toxicities of this agent preclude their utility as long-term maintenance therapy, as was the case with our patient.

As with breast cancer, expression of CDK4 has been reported as high in EMPD (in 94.1% of cells tested in vitro [4]). This suggested to us the use of CDK4/6 inhibitors, such as abemaciclib, as a potential therapy. We selected abemaciclib because it is effective as monotherapy, unlike the other CDK4/6 inhibitor currently available at the time this option was considered. It is generally well-tolerated and can be given without dose reduction in the setting of renal insufficiency. Our patient found the drug to be very well tolerated, and he was able to manage the side-effect of diarrhea very well. His course was monitored with serial PET/CT scans as well as a surrogate serum tumor marker CYFRA 21-1. Both of these suggested that the progression of the disease was controlled, and he has maintained a good quality of life with this drug. The patient presented survived four years with reasonable performance status after his progression to stage III EMPD due to an aggressive choice of agents, including abemaciclib. We suggest the use of this agent in patients with metastatic (and particularly) refractory EMPD.

Further, since the treatment of our patient, Kitamura et al. have reported pre-clinical data on the efficacy of abemaciclib in EMPD [6]. Mice bearing xenografted EMPD exhibited suppressed tumor growth and lowered Ki-67 expression after treatment with abemaciclib. This suggests the potential clinical benefit of abemaciclib, as we observed in our patient.

## Conclusions

Extramammary Paget's disease (EMPD) is a rare neoplasm in which relapse is common, and for which few agents have been identified as active, and with a toxicity profile suitable for long-term (maintenance) administration. Similarities with conventional breast cancer, including the overexpression of CDK4, suggested to us that abemaciclib might be suitable in this setting. Our patient’s metastatic disease was held stable for 16 months without evidence of progressive disease while being treated with abemaciclib. Despite the eventual development of refractory relapse, our patient with refractory and heavily pre-treated EMPD was maintained on abemaciclib for approximately two and half years with good quality of life.
